# The Role of TOMM40 in Cardiovascular Mortality and Conduction Disorders: An Observational Study

**DOI:** 10.3390/jcm13113177

**Published:** 2024-05-29

**Authors:** Giuseppe Di Stolfo, Sandra Mastroianno, Nicolò Soldato, Raimondo Salvatore Massaro, Giovanni De Luca, Davide Seripa, Maria Urbano, Carolina Gravina, Antonio Greco, Paola Siena, Marco Matteo Ciccone, Andrea Igoren Guaricci, Cinzia Forleo, Massimo Carella, Domenico Rosario Potenza

**Affiliations:** 1 Cardiovascular Department, Fondazione IRCCS Casa Sollievo della Sofferenza, 71013 San Giovanni Rotondo, FG, Italy; s.mastroianno@operapadrepio.it (S.M.); r.massaro@operapadrepio.it (R.S.M.); g.deluca@operapadrepio.it (G.D.L.); d.potenza@operapadrepio.it (D.R.P.); 2 University Cardiology Unit, Department of Interdisciplinary Medicine, Policlinic University Hospital, 70124 Bari, BA, Italy; n.soldato@operapadrepio.it (N.S.); p.siena@operapadrepio.it (P.S.); marcomatteo.ciccone@uniba.it (M.M.C.); andreaigoren.guaricci@uniba.it (A.I.G.); cinzia.forleo@uniba.it (C.F.); 3 Complex Structure of Geriatrics, Medical Sciences Department, Fondazione IRCCS Casa Sollievo della Sofferenza, 71013 San Giovanni Rotondo, FG, Italy; davide.seripa@asl.lecce.it (D.S.); m.urbano@operapadrepio.it (M.U.); c.gravina@operapadrepio.it (C.G.); a.greco@operapadrepio.it (A.G.); 4 Division of Medical Genetics, Fondazione IRCCS Casa Sollievo della Sofferenza, 71013 San Giovanni Rotondo, FG, Italy; m.carella@operapadrepio.it

**Keywords:** TOMM40, rs2075650, left bundle branch block, cardiac conduction disease, cardiac electronic device, cardiovascular death

## Abstract

**Aims**: TOMM40 single nucleotide polymorphism (SNP) rs2075650 consists of allelic variation c.275-31A > G and it has been linked to Alzheimer disease, apolipoprotein and cholesterol levels and other risk factors. However, data on its role in cardiovascular disorders are lacking. The first aim of the study is to evaluate mortality according to TOMM40 genotype in a cohort of selected patients affected by advanced atherosclerosis. Second aim was to investigate the relationship between Xg and AA alleles and the presence of conduction disorders and implantation of defibrillator (ICD) or pacemaker (PM) in our cohort. **Materials and Methods**: We enrolled 276 patients (mean age 70.16 ± 7.96 years) affected by hemodynamic significant carotid stenosis and/or ischemia of the lower limbs of II or III stadium Fontaine. We divided the population into two groups according to the genotype (Xg and AA carriers). We evaluated several electrocardiographic and echocardiographic parameters, including heart rate, rhythm, presence of right and left bundle branch block (LBBB and RBBB), PR interval, QRS duration and morphology, QTc interval, and left ventricular ejection fraction (LVEF). We clinically followed these patients for 82.53 ± 30.02 months and we evaluated the incidence of cardiovascular events, number of deaths and PM/ICD implantations. **Results:** We did not find a difference in total mortality between Xg and AA carriers (16.3 % vs. 19.4%; *p* = 0.62). However, we found a higher mortality for fatal cardiovascular events in Xg carriers (8.2% vs. 4.4%; HR = 4.53, 95% CI 1.179–17.367; *p* = 0.04) with respect to AA carriers. We noted a higher percentage of LBBB in Xg carriers (10.2% vs. 3.1%, *p* = 0.027), which was statistically significant. Presence of right bundle branch block (RBBB) was also higher in Xg (10.2% vs. 4.4%, *p* = 0.10), but without reaching statistically significant difference compared to AA patients. We did not observe significant differences in heart rate, presence of sinus rhythm, number of device implantations, PR and QTc intervals, QRS duration and LVEF between the two groups. At the time of enrolment, we observed a tendency for device implant in Xg carriers at a younger age compared to AA carriers (58.50 ± 0.71 y vs. 72.14 ± 11.11 y, *p* = 0.10). During the follow-up, we noted no statistical difference for new device implantations in Xg respect to AA carriers (8.2% vs. 3.5%; HR = 2.384, 95% CI 0.718–7.922; *p* = 0.156). The tendency to implant Xg at a younger age compared to AA patients was confirmed during follow-up, but without reaching a significant difference(69.50 ± 2.89 y vs. 75.63 ± 8.35 y, *p* = 0.074). Finally, we pointed out that Xg carriers underwent device implantation 7.27 ± 4.43 years before AA (65.83 ± 6.11 years vs. 73.10 ± 10.39 years) and that difference reached a statistically significant difference (*p* = 0.049) when we considered all patients, from enrollment to follow-up. **Conclusions**: In our study we observed that TOMM40 Xg patients affected by advanced atherosclerosis have a higher incidence of developing fatal cardiovascular events, higher incidence of LBBB and an earlier age of PM or ICD implantations, as compared to AA carriers. Further studies will be needed to evaluate the genomic contribution of TOMM40 SNPs to cardiovascular deaths and cardiac conduction diseases.

## 1. Introduction

The TOMM40 gene is located on chromosome 19 codifying Tom 40 protein, localized in the outer membrane of the mitochondria; it is the channel-forming subunit of the translocase of the mitochondrial outer membrane (TOM) complex, essential for import of protein precursors into mitochondria.

The most widely researched variants are rs2075650 and rs10524523, which have been linked to Alzheimer’s disease, longevity, total cholesterol levels, apolipoprotein expression, body mass index (BMI) and other cardiovascular risk factors [1,2,3,4,5,6]. The rs2075650 single nucleotide polymorphism (SNP) is situated within the non-coding region of the TOMM40 gene, and it consists of an allelic variation c.275-31A > G. The minor allele frequency (MAF) for the G allele is around 0.124. It is more prevalent in Europe and Africa (mean MAF: 0.131) and less prevalent in East Asian people (mean MAF: 0.092). Several genome-wide association studies (GWASs) have shown that allelic variations in the TOMM40 gene, specifically associated to G allele, may predispose to a variety of disorders, linked to lower BMI [7] and increased concentration of total cholesterol [3,8], worse cognitive function, altered inflammatory networks and higher susceptibility to vascular risk factors, whereas the A allele appears to be linked to higher longevity; this observation is not strictly related to TOMM40 mRNA transcript quantification or protein structure, but may be linked to a more complicated genetic regulatory network [9]. Nowadays, the clinical connection between TOMM40 genetic variants and Alzheimer’s disease is widely accepted. Indeed, the predisposition to increased deposition of beta-amyloid has been demonstrated [10]. Although numerous works have found a correlation between TOMM40 SNPs and cardiovascular risk factors, specific data on the role of TOMM40 SNPs in cardiovascular mortality and cardiac conduction disorders are lacking. An interesting hypothesis is that connexins (Cxs), which form the gap junctions at the cardiac cell-to-cell interface, are strongly related both to intercellular communication and to mitochondrial function. The aims of our study were firstly to investigate the relationship between TOMM40 genotypes and cardiovascular mortality, and secondly to evaluate the incidence of cardiac conduction disorders and implantation of defibrillators (ICD) or pacemakers (PM) in a cohort of patients with advanced atherosclerosis.

## 2. Material and Methods

We conducted a longitudinal observational study of a cardiovascular-controlled case series. The study protocol was approved by the Human Experimentation Ethics Committees of Casa Sollievo della Sofferenza Hospital (Ethics Committee code 150037, protocol name *TOMM40 versione 11 Gen 13* and date of approval 13 April 2013) and fulfilled the requirements of the Declaration of Helsinki, Strengthening the Reporting of Observational Studies in Epidemiology (STROBE) and the Guidelines for Good Clinical Practice.

We enrolled 276 patients (209 males and 67 females) from November 2009 to October 2017. Written informed consent was obtained from each patient. The inclusion criteria were Caucasian race and the presence of advanced atherosclerosis, defined as carotid plaques with hemodynamic significant stenosis evaluated by Doppler velocimetry measurement and/or II or III stadium Leriche–Fontaine claudication. Exclusion criteria were non-hemodynamically significant carotid atheroma, asymptomatic arterial disease (I stadium Fontaine), lower limb ischemia with gangrene (IV stadium Fontaine), and cancer with life expectancy less than six months. We evaluated clinical documentation of patients and collected information about history of cancer, cardiovascular disease (stroke and ischemic heart disease), hypertension, diabetes and dyslipidemia, according to criteria of World Health Organization and ATP III. All patients underwent complete blood count with cholesterol, triglycerides, glucose, homeostatic model assessment (HOMA-IR, calculated as fasting serum insulin (mUI/mL) × fasting plasma glucose (mmol/l)/22.5), creatinine and microalbuminuria levels. We measured blood pressure, waist and hip circumference and body mass index (BMI). All patients underwent echocardiographic evaluation with measurement of aortic bulb diameter, left atrium size, end-diastolic left ventricle dimensions, interventricular septum thickness, posterior wall diameter and left ventricular ejection fraction (LVEF) and ventricular mass index (LVMI). Electrocardiographic parameters, such as heart ratio, presence of LBBB and/or RBBB, PR, QRS and QTc intervals, were obtained by standard 12-lead electrocardiogram. Patients were clinically followed during a period of 82.53 ± 30.02 months (range 1–124). We recorded the followings: (i) major adverse cardiovascular events (MACE), defined as myocardial infarction, cerebral ischemia, myocardial and/or peripheral revascularization; (ii) total mortality, cancer deaths and cardiovascular mortality, which was defined as deaths caused by myocardial infarction, stroke or heart failure; (iii) PM or ICD implantations.

### 2.1. Genetic Analysis

AQ blood sample (2 mL) was taken from each patient and collected in EDTA-containing tubes. Genomic DNA was extracted from peripheral blood and genetic examination was performed from 2010 to 2017 as previously described. We studied the TOMM40 rs2075650 A>g polymorphism and found the following genotypic frequencies: 82.2% of AA, 16.7% of Ag and 1.1% of gg. No differences were observed with respect to the expected Hardy–Weinberg frequencies. According to these genotype frequencies, the estimated allele frequencies were 90.58% for the A allele and 9.42% for g allele. We divided patients in two groups: AA (AA) and Xg (Ag + gg). As AA was the most common genotype in the population, it was considered as “wild-type”, and patients with AA genotype were identified as the reference group in this study.

### 2.2. Statistical Analysis

All statistical analyses were performed with SPSS 25.0 software (Chicago, IL, USA). Continuous and categorical variables were presented as means ± standard deviation (SD) and frequency (%), respectively. Pearson’s χ^2^ test was used to compare dichotomous variables in the two groups. Hardy–Weinberg equilibrium was tested by χ^2^ test. Variance analysis (two-tailed unpaired *t* test) and Kolmogorov–Smirnov test were used to compare quantitative variables and verify normal distribution. Mann–Whitney test was used to compare age of events. *p* values < 0.05 were considered statistically significant. Cox model was applied to estimate device implantations, MACE and death incidence by hazard ratio (HR) with 95% confidence interval (95% CI). Kaplan–Meier curves were used to show events during follow up.

## 3. Results

### 3.1. Baseline Characteristics

In our outpatient clinic, we consecutively recruited 276 patients (209 males and 67 females, mean age 70.16 ± 7.96 years) affected by advanced atherosclerosis. Baseline clinical characteristics of the whole cohort divided according to TOMM40 genotype are listed in Table 1. At baseline, we observed that 17.5% of patients were normal weight (BMI between 20 and 25 kg/m^2^), 62% were overweight (BMI between 25 and 30 kg/m^2^), and 20.4% were obese (BMI ≥ 30 kg/m^2^). Waist circumference and waist-to-hip ratio were 100.35 ± 10.25 cm and 0.96 ± 0.07 cm, respectively. Compared to wild type, Xg patients had smaller waist circumference (97.75 ± 11.12 cm vs. 100.89 ± 10.0 cm; *p* = 0.064), lower waist to hip ratio (0.95 ± 0.08 vs. 0.97 ± 0.07; *p* = 0.13) and were less heavy (27.79 ± 3.87 vs. 28.55 ± 3.97; *p* = 0.22). In the two groups, we found no statistically significant differences in blood pressure, fasting blood glucose and HOMA-IR. With respect to AA, Xg carriers showed a tendency to have lower levels of triglycerides (117.21 ± 58.89 mg/dL vs. 122.27 ± 58.86 mg/dL; *p* = 0.79), total cholesterol (162.92 ± 37.54 mg/dL vs. 167.29 ± 42.26 mg/dL; *p* = 0.51), LDL cholesterol (90.16 ± 32.58 mg/dL vs. 94.73 ± 36.35 mg/dL; *p* = 0.42) and microalbuminuria (39.18 ± 127.83 µg/min vs. 58.72 ± 127.24 µg/min; *p* = 0.10). We did not observe differences in PR, QRS and QTc intervals and presence of sinus rhythm between the two groups. The mean values of diameters of aortic bulb, left atrium, end-diastolic left ventricle, thickness of interventricular septum and posterior wall of left ventricle, LVEF, LVMI were not statistically different in the two groups.

We present a description of comorbidities and medical treatments at baseline in Table 2. In our cohort, patients had 86.6% hypertension, 80.9% dyslipidemia, 44.2% diabetes, 37.9% ischemic heart disease and 14.5% stroke. They presented a smoking habit in 21.4% of cases and had a history of neoplasia in 14.5% of cases. According to TOMM40 genotype, no significant differences of hypertension, dyslipidemia, type 2 diabetes, smoking habit, ischemic heart disease, stroke and cancer were observed between the two groups at the baseline. Furthermore, no significant differences in medical treatments were observed between the two groups. Concerning cardiac conduction diseases, Xg carriers showed a higher incidence of LBBB compared to wild type (10.2% vs. 3.1%; *p* = 0.027), and this difference was statistically significant. The incidence of right bundle branch block (RBBB) also appeared more prevalent in Xg carriers (10.2% vs. 4.4%; *p* = 0.10), but this was not statistically significant.

At baseline, twenty-three patients had cardiac devices (16 pacemakers and 7 defibrillators), particularly, 2 Xg carriers and 21 AA carriers (4.4% vs. 9.6%). In this case, we did not register a statistically significant difference (*p* = 0.27). Instead, we observed a tendency for device implantation in Xg carriers at a younger age compared to AA carriers (58.50 ± 0.71 y vs. 72.14 ± 11.11 y, *p* = 0.10).

### 3.2. Outcomes

**Figure 1:**
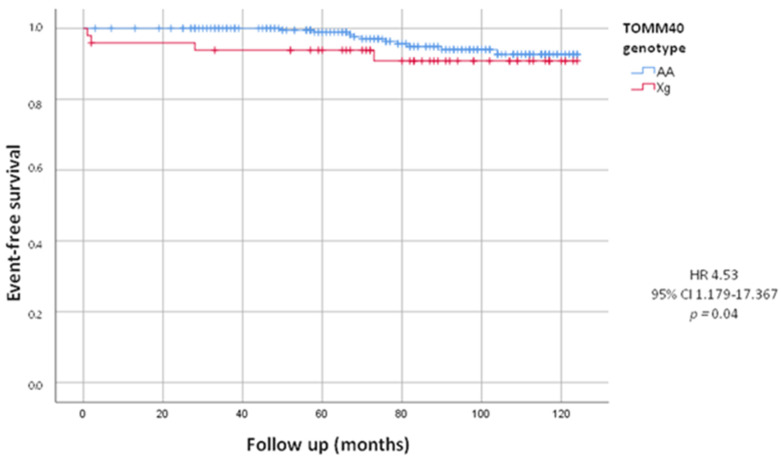
Kaplan-Meier curves comparing cardiovascular death stratified by TOMM40 genotype. The test comparing the two groups was based on the log-rank test. The curves showed that Xg group (red line) had significantly higher mortality from cardiovascular events respect to AA group (blue line).

After a follow-up of 82.53 ± 30.02 months, we evaluated total combined incidence of deaths, MACE and PM/ICD implantations, as presented in Table 3. During the follow-up we recorded 65 (23.6%) MACE. We did not observe a statistically significant difference between the Xg and AA group (22.4% vs. 23.8%, HR = 0.94, 95% CI 0.493–1.802; *p* = 0.86). We also recorded 52 deaths, including 14 by cardiovascular events, 17 by cancers and 21 by other causes. We did not observe a statistically significant difference in deaths from all causes between the two groups (16.3% in Xg group vs. 19.4% in AA group, HR = 0.84, 95% CI 0.395–1.784; *p* = 0.62). The mean age of death was 78.46 ± 9.92 years, in detail 79.38 ± 5.04 years in Xg group and 78.30 ± 7.25 years in AA group (*p* = 0.63). Woth respect to AA, we found a higher mortality, statistically significant, from cardiovascular events in Xg carriers (8.2% vs. 4.4%; HR = 4.53, 95% CI 1.179–17.367; *p* = 0.04) with no difference of age (82.0 ± 5.29 y vs. 81.90 ± 8.34 y, *p* = 0.84). In Figure 1, Kaplan–Meier’s survival curves for cardiovascular deaths according to TOMM40 genotype were shown.

With respect to wild type, in Xg carriers we no found difference of cancer death (4.1% vs. 6.6%; HR = 1.79, 95% CI 0.387–8.265; *p* = 0.46) and age of death (74.50 ± 0.71 y vs. 74.80 ± 5.44 y, *p* = 0.76), as shown in Table 3.

**Figure 2:**
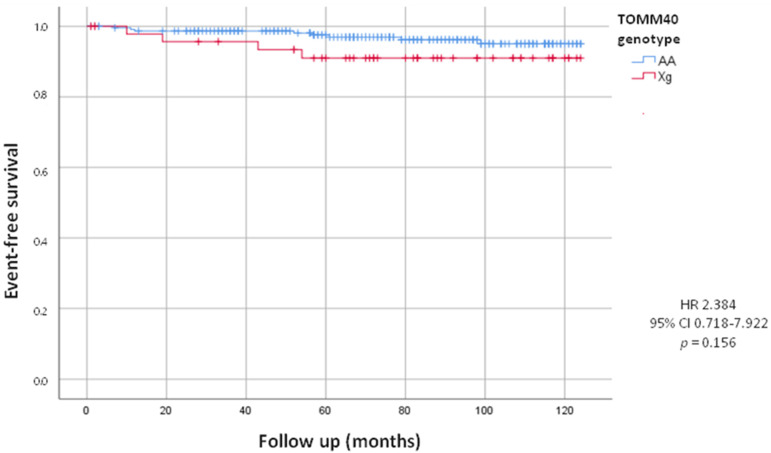
Kaplan-Meier curves comparing cardiac device implants stratified by TOMM40 genotype. The test comparing the two groups was based on the log-rank test. The curves show the trend of cardiac device implants in the Xg group (red line) and in the AA group (blue line).

On the other hand, during the follow up, we observed that twelve patients underwent device implantation (8 PM and 4 ICD). Prospective analysis showed no statistically significant difference in the incidence of PM and ICD implants between Xg and AA carriers (8.2% of Xg vs. 3.5% of AA; HR = 2.384, 95% CI 0.718–7.922; *p* = 0.156), although we can observe a tendency in Xg group (Figure 2).

Table 4 showed the number and the age of PM/ICD implants at baseline, during the follow-up and the full amount according to TOMM40 genotype. At the end of the observational period, the total sum of patients with a cardiac device (previously implanted and during the follow-up) was 35 (12.7%), particularly 6 Xg (12.2%) and 29 AA carriers (12.8%), and that difference was not statistically significant. On the other hand, the tendency observed at the baseline to implant Xg at a younger age with respect to AA carriers was confirmed during follow-up (69.50 ± 2.89 y vs. 75.63 ± 8.35 y, *p* = 0.074). However, this tendency reached statistical significance when we considered all patients who had the device already implanted at enrolment and during the follow-up. Indeed, we recorded a mean implant age of 72.22 ± 10.22 years, but we also observed that Xg carriers underwent implantation 7.27 ± 4.43 years before AA carriers (65.83 ± 6.11 years vs. 73.10 ± 10.39 years) and that this difference was statistically significant (*p* = 0.049), as shown by the representation of difference of the age of PM/ICD implantations according to TOMM40 genotype in Table 4.

## 4. Discussion

According to our knowledge, this is the first observational clinical study that attempted to investigate the TOMM40 genetic variants in cardiovascular and cardiac conduction disorders. Our results showed that haplotypes in the TOMM40 rs2075650 chromosomal region might play an interesting role in cardiovascular death, higher incidence of LBBB and device implantations.

It is well known that mitochondria represent the energy cellular base because one of their main functions is to produce ATP [11]. However, several studies show that mitochondria are also important in regulating cellular metabolism and signaling through apoptosis processes [12,13,14,15,16]. Appropriate function of mitochondria is crucial for cardiovascular system cells (including cardiomyocytes, smooth muscle cells, fibroblasts and endothelial cells), which depends on elevated oxygen and metabolic supply to function properly.

Currently, the role of mitochondrial dynamics and protein import networks represents a key determinant for respiratory chain malfunction and mitochondrial reactive oxygen species (ROS), suggesting potential novel routes in cardiovascular disorders.

In this context, the TOM complex plays a fundamental role because it represents the major import system of pre-proteins within mitochondria [17,18,19].

The central pore of the TOM complex is formed by TOMM40, TOMM22 and the associated subunits TOM5, TOM6 and TOM7. Tom40 is a 19-stranded beta-barrel protein with largely negatively charged regions and one positively charged area close to the intermembrane space [20,21,22,23,24].

It is known that mitochondrial DNA mutations can lead to a variety of genetic disorders that may be systemic or cardio-specific, even if the real mechanism still remains unclear; In any case, data regarding the role of mitochondrial outer and/or inner membrane proteins in cardiovascular diseases are lacking.

To this point, the TOMM40 SNP rs2075650 has been studied in several works highlighting its critical role in mitochondrial dysfunction in Alzheimer’s disease and cardiac preconditioning, by both impairing mitochondrial Ca^2+^ signaling and mtPTP (mitochondrial permeability transition pore) opening [25,26,27,28,29,30,31].It is known that different cells of the cardiovascular system (cardiomyocytes, smooth muscle cells, fibroblasts and endothelial cells) depend on the proper functioning of the mitochondrial chain machinery [32]. The heart requires high energy levels to function properly, thus it is clear that mitochondrial protein import disfunction may result in cardiovascular disorders.

However, only few studies have investigated the role of the TOM complex subunits in cardiac diseases; Boengler pointed out the first findings from an in vivo model in 2006 that demonstrated reduced TOMM20 expression after ischemia damage in isolated mitochondria from pig hearts, highlighting the important role of this protein in mechanisms of ischemic pre-conditioning [33].

Further, Zhang et al. recently showed that TOMM22 interact with mitochondrial calcium-activated potassium (BKCa) cardiac channels; this might imply that the TOM complex has a physiological role in Ca^2+^ import by cardiac mitochondria, therefore a TOM22 deficiency may compromise cardiac cell physiology [34,35].

On the other hand, the TOMM40 SNPrs2075650 A and G alleles are the most commonly analyzed variations. A recent review from Chen et al. [9] reported that the major allele A has a prediction of survival over 90 years in Chinese, European, white, black and Hispanic populations [36,37,38,39,40], whereas the minor allele G is linked to impaired cognitive performance, altered inflammatory networks and higher predisposition to vascular risk factors and lower BMI [7,41,42,43,44,45].

In our study, we demonstrated for the first time a clinical correlation between TOMM40 Xg carriers and an increased risk of cardiovascular disease, expressed in terms of higher cardiovascular mortality and higher incidence of cardiac conduction diseases, represented by greater incidence of LBBB and earlier age of PM/ICD implantation.

Several pathophysiological mechanisms can be hypothesized to explain these results. Firstly, we found that the G allele predisposes to higher cardiovascular mortality with respect to A carriers. Patients with G variant had a lower BMI with respect to A variant, in line with the analysis of Chen et al. However, this finding did not reach statistical significance in our patient cohort, most likely due to a lower sample size. Furthermore, we did not observe a difference in all-cause mortality between the two groups, but we found that Xg patients had a higher incidence of cardiovascular death with respect to A carriers. It has already been demonstrated that rs2075650-g is associated with lower BMI, higher total cholesterol, apolipoprotein B and E concentration [3,7,8,46,47]. Additionally, Gui et al. [45] reported that cardiovascular risk factors, including current smoking, drinking, inactivity, obesity, total cholesterol, triglycerides, high lipoprotein cholesterol, low-density lipoprotein cholesterol, diabetes and hypertension, may work synergistically with the rs2075650-gvariant. These findings may explain the higher cardiovascular mortality, reinforcing the hypothesis that the Xg allele variant plays a role in increased predisposition to cardiovascular diseases and higher susceptibility to cardiac risk factors, even in a very high-risk population like that of our patients, suggesting a new role of TOMM40 variant determining residual cardiovascular risk. Cardiac mortality may be worsened by impaired ischemic preconditioning and subsequent increased myocardial infarction extension after coronary occlusion.

On the other hand, we showed an association between TOMM40 Xg polymorphism and higher LBBB incidence, suggesting a role of mitochondria machinery in cardiac conduction diseases. Cardiomyocytes’ synchronized contraction requires fast electrical excitation propagation. Thus, cardiac action potential moves across the cardiomyocytes through low-resistance channels at the cell-to-cell interfaces, represented by the gap junctions [48]. Cardiac gap junctions are composed of connexins (Cxs), that enable electrical coupling by intercellular electrical and metabolic communication [49,50,51,52]. Indeed, they guarantee proper cardiac rhythm maintenance, control of vascular tone, endothelial function and metabolic exchange between nearby cells [53]. In the normal adult heart, three main isoforms are expressed: Cx40, Cx43 and Cx45. Defects in the expression of cardiac connexins may lead to aberrant activation of the cardiac fibers and result in conduction diseases. The role of cardiac Cxs in cardiac conduction block was confirmed for the first time by Makita et al. [54], which underlined the importance of Cx40 in the correct propagation of the electrical impulse, since the heterologous expression of Cx40-Q58L mutations may cause familial heart block. Particularly, Cx43 is most expressed in the atrial and ventricular myocytes [55]. In neo-natal rat heart cells, pharmacological blockade of gap junctional channels with octanol or palmitoleic acid decreased ventricular conduction velocity [56], whereas octanol treatment in pig papillary muscles caused a slower propagation of action potentials before complete conduction block [57].

Studies using co-immunoprecipitation have revealed a connection between Cx43 and TOMM20, which is an important component of the TOM complex. This could explain that TOMM40, paired to TOMM20, may lead to TOM complex impairment affecting Cx43 ability to work properly. Although the Cx43 physiological function has not been fully explored, new studies indicate that it regulates K+ inflow to the mitochondrial matrix, mitochondrial respiration, and the production of reactive oxygen species (ROS) [58]. It has been demonstrated that cardiac conduction velocity decreases by 50% when Cx43 expression is reduced by around 90%, and this observation supports the hypothesis of our data [59]. It has also been shown that a reduction in excitability by sodium current amplitude results from loss of Cx43 expression, thus resulting in disruption of the cell-to-cell signaling pathway and reduction in the differential voltage produced by stimulated cells [60].

All these experimental studies open an interesting scenario about the role of TOM complex in connexins regulation, supporting the hypothesis of a “connexin connection” and suggesting a plausible role for TOMM40 SNP in cardiac conduction disease in our study; since Cx43, among other functions, regulates the correct propagation of cardiac impulse, and the Cx43 concentration depends on correct import into mitochondria through TOM23, we can speculate that TOMM40 deficiency may induce a dysfunction of the entire TOM complex with Cx43 downregulation.

Currently, it is clear that several physio-pathological events induce atrial and intraventricular conduction abnormalities. These phenomena are not just age-related but they are often molecularly linked. The control of the electrical signal is governed by a large number of genes and proteins, and only a small subset of genetic variants has been identified. In this observational study, we showed a link between the allelic variation of TOMM40 and a clinical evidence of higher prevalence of LBBB and PM/ICD implantation at younger age. Future research will be needed, based on both new cellular models and larger population study, in order to establish the direct cause-effect relationship between the genetic variations and the impact of cardiac conduction disorders, moving from our results linking TOMM40 SNP to higher cardiovascular mortality.

Based on our data, we believe that TOMM40 should be included in the polygenic risk score (PRS) factor for cardiovascular disease prevention in the future.

Different patient management and treatment models may be improved, allowing a better personalized care approach oriented to improving mortality and quality of life.

Firstly, we observed a residual cardiovascular risk in Xg population, even in a very high-risk population; this assumption may imply a more aggressive treatment (e.g., lipid lowering therapy) and a closer follow up. Secondly, the conduction disease, possibly associated also with ventricular arrhythmia, based on conduction delay caused by more fibrosis and scar channel reentry induced by connexin dysfunction, may lead to a different disease management, through loop recorder implantation for adequate and early arrhythmias detection. Lastly, a worse ischemic preconditioning may lead to a greater scar after myocardial infarction in Xg carriers, leading to heart failure and cardiac arrhythmias; further studies need to explore the role of pharmacological preconditioning in this higher risk population.

## 5. Study Limitation

Although our study contains many suggestions regarding the role of TOMM40 variation in cardiac conduction disease and MACE, the sample size may represent a limit; at the same time, our particular population, constituted of very high-risk old patients, may limit the result extension to the general population. Furthermore, the evaluated population origins from a specific region, in North Apulia (Southern Italy), characterized by the Mediterranean diet and low level of physical activity, yet we did not collect specific daily activity amounts and data on detailed diet; these may represent confounding factors.

## 6. Conclusions

In our study, we showed that TOMM40 Xg patients with advanced atherosclerosis have a higher incidence of LBBB, an earlier age of PM or ICD implantation and a higher percentage of fatal cardiovascular deaths with respect to AA carriers. Currently, only few studies have examined the cardiac effects associated with this allelic variant. Our result opens up a fascinating scenario on the relationship between TOMM40 SNPs and conduction disease, ischemic preconditioning and arrhythmia burden, beyond the classic cardiovascular risk factors. Understanding the TOM complex machinery and its impact on cardiac diseases may help physicians to develop specific genetic drugs that target the TOM40 complex. Of note, further research will elucidate this issue and help us to better understand the complexity of genetic interaction of the TOM complex in cardiovascular diseases.

## Figures and Tables

**Table 1:** Baseline clinical characteristics of the whole cohort according to TOMM40 genotype.

	All Patients	TOMM40 Genotypes		
		AA	Xg	*p* Value
Number of subjects	276	227	49	
Age (y)	70.16 ± 7.96	69.98 ± 7.80	71.0 ± 8.72	0.42
Gender (male/female)	209/67	176/33	51/16	0.13
BMI (kg/m^2^)	28.42 ± 3.95	28.55 ± 3.97	27.79 ± 3.87	0.22
Waist circumference (cm)	100.35 ± 10.25	100.89 ± 10.0	97.75 ± 11.12	0.064
Waist-hip ratio	0.96 ± 0.07	0.97 ± 0.07	0.95 ± 0.08	0.13
Systolic blood pressure (mmHg)	132.95 ± 17.22	133.04 ± 17.62	132.50 ± 15.36	0.87
Diastolic blood pressure (mmHg)	79.25 ± 6.64	79.39 ± 6.44	78.53 ± 7.62	0.49
Pulse pressure (mmHg)	53.71 ± 14.96	53.65 ± 15.56	53.97 ± 11.78	0.95
Fasting glucose (mg/dL)	117.22 ± 37.88	117.49 ± 36.79	116.00 ± 42.87	0.80
HOMA-IR	4.74 ± 7.17	4.81 ± 7.45	4.39 ± 5.75	0.66
Triglycerides (mg/dL)	121.36 ± 57.15	122.27 ± 58.86	117.21 ± 58.89	0.79
Total Ch (mg/dL)	166.51 ± 41.42	167.29 ± 42.26	162.92 ± 37.54	0.51
HDL-Ch (mg/dL)	49.35 ± 12.38	49.31 ± 12.56	49.52 ± 12.62	0.91
LDL-Ch (mg/dL)	93.91 ± 35.69	94.73 ± 36.35	90.16 ± 32.58	0.42
Serum creatine (mg/dL)	1.03 ± 0.58	1.03 ± 0.62	1.01 ± 0.33	0.87
Microalbuminuria (µg/min)	55.30 ± 127.30	58.72 ± 127.24	39.18 ± 127.83	0.10
Sinus rhythm (n, %)	262 (94.9%)	215 (94.7%)	47 (95.9%)	0.73
Right bundle branch block (n, %)	15 (5.4%)	10 (4.4%)	5 (10.2%)	0.10
Left bundle branch block (n, %)	12 (4.3%)	7 (3.1%)	5 (10.2%)	0.027
Implantation age of cardiac device (y)	70.96 ± 11.30	72.14 ± 11.11	58.50 ± 0.71	0.10
PR interval (ms)	162.74 ± 27.42	163.26 ± 28.44	160.32 ± 22.21	0.55
QRS interval (ms)	100.16 ± 21.20	100.05 ± 28.44	100.68 ± 21.78	0.87
QTc interval (ms)	415.58 ± 23.54	414.58 ± 23.75	414.58 ± 22.78	1.00
Aortic bulb diameter (mm)	33.07 ± 4.24	33.0 ± 4.13	33.34 ± 4.75	0.62
Left atrium diameter (mm)	35.52 ± 5.72	35.6 ± 5.74	35.19 ± 5.65	0.66
End-diastolic left ventricle (mm)	46.01 ± 6.25	45.96 ± 6.34	46.27 ± 5.86	0.75
Interventricular septum (mm)	11.76 ± 1.24	11.76 ± 1.26	11.75 ± 1.14	0.97
Posterior wall (mm)	11.13 ± 1.04	11.71 ± 1.03	11.11 ± 1.08	0.87
Heart rate (bpm)	70.0 ± 10.67	70.41 ± 10.52	68.27 ± 11.20	0.21
LVEF (%)	58.75 ± 5.61	58.64 ± 5.47	59.23 ± 6.23	0.51
LVMI (gr/m^2^)	76.29 ± 20.22	76.39 ± 20.71	75.84 ± 17.96	0.88

Continuous data are presented as means ± standard deviation (SD); categorical data are presented as number and frequency (%). BMI: body mass index; bpm: beats per minute; Ch: cholesterol; HOMA-IR: homeostatic model assessment; LVEF: left ventricular ejection fraction; LVMI: ventricular mass index; y: years.

**Table 2:** Description of comorbidities and medical treatments at baseline of the whole cohort according to TOMM40 genotype.

	All Patients		TOMM40 Genotypes				
			AA		Xg		*p* Value
**Comorbidities**							
Hypertension	239	(86.6%)	197	(86.8%)	42	(85.7%)	0.84
Dyslipidemia	220	(80.9%)	180	(80.7%)	40	(81.6%)	0.88
Type 2 diabetes	122	(44.2%)	103	(45.4%)	19	(38.8%)	0.40
Smoking	59	(21.4%)	49	(21.6%)	10	(20.4%)	0.88
Cardiac device implantation	23	(8.7%)	21	(9.6%)	2	(4.4%)	0.27
Ischemic heart disease	102	(37.9%)	86	(37.9%)	16	(32.7%)	0.49
Stroke	40	(14.5%)	31	(13.7%)	9	(18.4%)	0.39
Cancer	40	(14.5%)	35	(15.4%)	5	(10.2%)	0.35
**Medical treatments**							
ARBs	105	(38.0%)	86	(37.9%)	19	(38.8%)	0.91
ACE inhibitors	103	(37.3%)	86	(37.9%)	17	(34.7%)	0.67
Calcium channel blockers	72	(26.1%)	58	(25.6%)	14	(28.6%)	0.66
β-blockers	77	(26.4%)	60	(26.4%)	17	(27.9%)	0.26
Diuretics	120	(43.5%)	100	(44.1%)	20	(40.8%)	0.68
Antiplatelet	246	(89.1%)	205	(90.3%)	41	(83.7%)	0.18
Lipid-lowering drugs	2242	(87.7%)	197	(86.8%)	45	(91.8%)	0.33
Antidiabetic therapy	90	(32.6%)	75	(33.0%)	15	(30.6%)	0.74

Data are presented as number (%) of subjects. ACE inhibitors: angiotensin converting enzyme inhibitors; ARBs: angiotensin receptor blockers.

**Table 3:** Cardiovascular events (MACE and device implantations) and fatal events at follow-up according to TOMM40 genotype.

All Patients		TOMM40 Genotypes			
		AA	Xg	HR (95% CI)	*p* Value
**Cardiovascular events**					
MACE	65 (23.6%)	54 (23.8%)	11 (22.4%)	0.94 (0.493–1.802)	0.86
Cardiac device implantation	12 (4.3%)	8 (3.5%)	4 (8.2%)	2.384 (0.718–7.922)	0.156
**Fatal events**					
Total death	52 (18.8%)	44 (19.4%)	8 (16.3%)	0.84 (0.395–1.784)	0.62
Cardiovascular death	14 (5.1%)	10 (4.4%)	4 (8.2%)	4.53 (1.179–17.367)	0.04
Cancer death	17 (6.2%)	15 (6.6%)	2 (4.1%)	1.79 (0.387–8.265)	0.46

Data are presented as number (%) of subjects. MACE: Major adverse cardiovascular events.

**Table 4:** Number and age of device implantation at baseline, during follow-up and total of the whole cohort according to TOMM40 genotype.

	All Patients	TOMM40 Genotypes		
		AA	Xg	*p* Value
**T0 (time of enrollment)**	Number of implants	21 (9.6)	2 (4.4%)	0.27
	Age of implantation	72.14 ± 11.11	58.50 ± 0.71	0.10
**During follow-up**	Number of implants	8 (3.5%)	4 (8.2%)	0.156
	Age of implantation	75.63 ± 8.35	69.50 ± 2.89	0.074
**Total**	Number of implants	29 (12.8)	6 (12.2)	0.919
	Age of implantation	73.10 ± 10.39	65.83 ± 6.11	0.049

## Data Availability

The original contributions presented in the study are included in the article, further inquiries can be directed to the corresponding author.

## Abbreviations

BMI	body mass index
Cx	connexin
ICD	implantable cardiac defibrillator
LBBB	left bundle branch block
LVEF	left ventricle ejection fraction
MACE	major adverse clinical event
MAF	minor allele frequency
PM	pacemaker
RBBB	right bundle branch block
SNP	single nucleotide polymorphism
TOMM40	translocase of outer mitochondrial membrane 40

## References

[1] Johnson S.C., La Rue A., Hermann B.P., Xu G., Koscik R.L., Jonaitis E.M., Bendlin B.B., Hogan K.J., Roses A.D., Saunders A.M. (2011). The effect of TOMM40 poly-T length on gray matter volume and cognition in middle-aged persons with APOE ε3/ε3 genotype. Alzheimers Dement..

[2] Lutz M.W., Crenshaw D.G., Saunders A.M., Roses A.D. (2010). Genetic variation at a single locus and age of onset for Alzheimer's disease. Alzheimers Dement..

[3] Aulchenko Y.S., Ripatti S., Lindqvist I., Boomsma D., Heid I.M., Pramstaller P.P., Penninx B.W.J.H., Janssens A.C.J.W., Wilson J.F., Spector S.T. (2009). Loci influencing lipid levels and coronary heart disease risk in 16 European population cohorts. Nat. Genet..

[4] Middelberg R.P., Ferreira M.A., Henders A.K., Heath A.C., Madden P.A., Montgomery G.W., Martin N.G., Whitfield J.B. (2011). Genetic variants in *LPL*, *OASL* and *TOMM40*/*APOE-C1-C2-C4* genes are associated with multiple cardiovascular-related traits. BMC Med. Genet..

[5] Sandhu M.S., Waterworth D.M., Debenham S.L., Wheeler E., Papadakis K., Zhao J.H., Song K., Yuan X., Johnson T., Ashford S. (2008). LDL-cholesterol concentrations: A genome-wide association study. Lancet.

[6] Zhang Z., Tao L., Chen Z., Zhou D., Kan M., Zhang D., Li C., He L., Liu Y. (2011). Association of Genetic Loci with Blood Lipids in the Chinese Population. PLOS ONE.

[7] Guo Y., Lanktree M.B., Taylor K.C., Hakonarson H., Lange L.A., Keating B.J. (2013). Gene-centric meta-analyses of 108 912 individuals confirm known body mass index loci and reveal three novel signals. Hum. Mol. Genet..

[8] Salakhov R.R., Goncharovaa I.A., Makeeva O.A., Golubenko M.V., Kulish E.V., Kashtalap V.V., Barbarash O.L., Puzyrev V.P. (2014). [TOMM40 gene polymorphism association with lipid profile]. Genetika.

[9] Chen S., Sarasua S.M., Davis N.J., DeLuca J.M., Boccuto L., Thielke S.M., Yu C.E. (2022). TOMM40 genetic variants associated with healthy aging and longevity: A systematic review. BMC Geriatr..

[10] Cruchaga C., Nowotny P., Kauwe J.S., Ridge P.G., Mayo K., Bertelsen S., Hinrichs A., Fagan A.M., Holtzman D.M., Morris J.C. (2011). Association and expression analyses with single-nucleotide polymorphisms in TOMM40 in Alzheimer disease. Arch. Neurol..

[11] Pfanner N., Warscheid B., Wiedemann N. (2019). Mitochondrial proteins: From biogenesis to functional networks. Nat. Rev. Mol. Cell Biol..

[12] Park C.B., Larsson N.G. (2011). Mitochondrial DNA mutations in disease and aging. J. Cell Biol..

[13] Paradies G., Ruggiero F.M., Petrosillo G., Quagliariello E. (1997). Age-dependent decline in the cytochrome c oxidase activity in rat heart mitochondria: Role of cardiolipin. FEBS Lett..

[14] Voos W. (2013). Chaperone-protease networks in mitochondrial protein homeostasis. Biochim. Biophys. Acta.

[15] Quirós P.M., Langer T., López-Otín C. (2015). New roles for mitochondrial proteases in health, ageing and disease. Nat. Rev. Mol. Cell Biol..

[16] Wallace D.C. (2005). A mitochondrial paradigm of metabolic and degenerative diseases, aging, and cancer: A dawn for evolutionary medicine. Annu. Rev. Genet..

[17] Palmer C.S., Anderson A.J., Stojanovski D. (2021). Mitochondrial protein import dysfunction: Mitochondrial disease, neurodegenerative disease and cancer. FEBS Lett..

[18] Chacinska A., Koehler C.M., Milenkovic D., Lithgow T., Pfanner N. (2009). Importing mitochondrial proteins: Machineries and mechanisms. Cell.

[19] Kang Y., Baker M.J., Liem M., Louber J., McKenzie M., Atukorala I., Ang C.S., Keerthikumar S., Mathivanan S., Stojanovski D. (2016). Tim29 is a novel subunit of the human TIM22 translocase and is involved in complex assembly and stability. Elife.

[20] Pitt A.S., Buchanan S.K. (2021). A Biochemical and Structural Understanding of TOM Complex Interactions and Implications for Human Health and Disease. Cells.

[21] Guan Z., Yan L., Wang Q., Qi L., Hong S., Gong Z., Yan C., Yin P. (2021). Structural insights into assembly of human mitochondrial translocase TOM complex. Cell Discov..

[22] Armstrong L.C., Saenz A.J., Bornstein P. (1999). Metaxin 1 interacts with metaxin 2, a novel related protein associated with the mammalian mitochondrial outer membrane. J. Cell Biochem..

[23] Taylor R.D., McHale B.J., Nargang F.E. (2003). Characterization of Neurospora crassa Tom40-deficient mutants and effect of specific mutations on Tom40 assembly. J. Biol. Chem..

[24] Wang W., Chen X., Zhang L., Yi J., Ma Q., Yin J., Zhuo W., Gu J., Yang M. (2020). Atomic structure of human TOM core complex. Cell Discov..

[25] Bogorodskiy A., Okhrimenko I., Burkatovskii D., Jakobs P., Maslov I., Gordeliy V., Dencher N.A., Gensch T., Voos W., Altschmied J. (2021). Role of Mitochondrial Protein Import in Age-Related Neurodegenerative and Cardiovascular Diseases. Cells.

[26] Puschmann A. (2013). Monogenic Parkinson’s disease and parkinsonism: Clinical phenotypes and frequencies of known mutations. Parkinsonism Relat. Disord..

[27] Kasten M., Hartmann C., Hampf J., Schaake S., Westenberger A., Vollstedt E.J., Balck A., Domingo A., Vulinovic F., Dulovic M. (2018). Genotype-Phenotype Relations for the Parkinson’s Disease Genes Parkin, PINK1, DJ1: MDSGene Systematic Review. Mov. Disord..

[28] Di Maio R., Barrett P.J., Hoffman E.K., Barrett C.W., Zharikov A., Borah A., Hu X., McCoy J., Chu C.T., Burton E.A. (2016). α-Synuclein binds to TOM20 and inhibits mitochondrial protein import in Parkinson’s disease. Sci. Transl. Med..

[29] Swerdlow R.H., Burns J.M., Khan S.M. (2014). The Alzheimer’s disease mitochondrial cascade hypothesis: Progress and perspectives. Biochim. Biophys. Acta.

[30] Cenini G., Rüb C., Bruderek M., Voos W. (2016). Amyloid β-peptides interfere with mitochondrial preprotein import competence by a coaggregation process. Mol. Biol. Cell.

[31] Hansson Petersen C.A., Alikhani N., Behbahani H., Wiehager B., Pavlov P.F., Alafuzoff I., Leinonen V., Ito A., Winblad B., Glaser E. (2008). The amyloid beta-peptide is imported into mitochondria via the TOM import machinery and localized to mitochondrial cristae. Proc. Natl. Acad. Sci. USA.

[32] Heinemeyer T., Stemmet M., Bardien S., Neethling A. (2019). Underappreciated Roles of the Translocase of the Outer and Inner Mitochondrial Membrane Protein Complexes in Human Disease. DNA Cell Biol..

[33] Boengler K., Gres P., Cabestrero A., Ruiz-Meana M., Garcia-Dorado D., Heusch G., Schulz R. (2006). Prevention of the ischemia-induced decrease in mitochondrial Tom20 content by ischemic preconditioning. J. Mol. Cell Cardiol..

[34] Sakaue H., Shiota T., Ishizaka N., Kawano S., Tamura Y., Tan K.S., Imai K., Motono C., Hirokawa T., Taki K. (2019). Porin Associates with Tom22 to Regulate the Mitochondrial Protein Gate Assembly. Mol. Cell.

[35] Zhang J., Li M., Zhang Z., Zhu R., Olcese R., Stefani E., Toro L. (2017). The mitochondrial BK(Ca) channel cardiac interactome reveals BK(Ca) association with the mitochondrial import receptor subunit Tom22, and the adenine nucleotide translocator. Mitochondrion.

[36] Deelen J., Beekman M., Uh H.W., Broer L., Ayers K.L., Tan Q., Kamatani Y., Bennet A.M., Tamm R., Trompet S. (2014). Genome-wide association meta-analysis of human longevity identifies a novel locus conferring survival beyond 90 years of age. Hum. Mol. Genet..

[37] Yashin A.I., Arbeev K.G., Wu D., Arbeeva L.S., Bagley O., Stallard E., Kulminski A.M., Akushevich I., Fang F., Wojczynski M.K. (2018). Genetics of Human Longevity From Incomplete Data: New Findings From the Long Life Family Study. J. Gerontol. A Biol. Sci. Med. Sci..

[38] Shadyab A.H., Kooperberg C., Reiner A.P., Jain S., Manson J.E., Hohensee C., Macera C.A., Shaffer R.A., Gallo L.C., LaCroix A.Z. (2017). Replication of Genome-Wide Association Study Findings of Longevity in White, African American, and Hispanic Women: The Women’s Health Initiative. J. Gerontol. A Biol. Sci. Med. Sci..

[39] Lin R., Zhang Y., Yan D., Liao X., Gong G., Hu J., Fu Y., Cai W. (2016). Association of common variants in TOMM40/APOE/APOC1 region with human longevity in a Chinese population. J. Hum. Genet..

[40] Lu F., Guan H., Gong B., Liu X., Zhu R., Wang Y., Qian J., Zhou T., Lan X., Wang P. (2014). Genetic variants in PVRL2-TOMM40-APOE region are associated with human longevity in a Han Chinese population. PLoS ONE.

[41] Arpawong T.E., Pendleton N., Mekli K., McArdle J.J., Gatz M., Armoskus C., Knowles J.A., Prescott C.A. (2017). Genetic variants specific to aging-related verbal memory: Insights from GWASs in a population-based cohort. PLoS ONE.

[42] Li T., Pappas C., Le S.T., Wang Q., Klinedinst B.S., Larsen B.A., Pollpeter A., Lee L.Y., Lutz M.W., Gottschalk W.K. (2022). APOE, TOMM40, and sex interactions on neural network connectivity. Neurobiol. Aging.

[43] Kulminski A.M., Loika Y., Culminskaya I., Huang J., Arbeev K.G., Bagley O., Feitosa M.F., Zmuda J.M., Christensen K., Yashin A.I. (2019). Independent associations of TOMM40 and APOE variants with body mass index. Aging Cell.

[44] Lamparello A.J., Namas R.A., Schimunek L., Cohen M., El-Dehaibi F., Yin J., Barclay D., Zamora R., Billiar T.R., Vodovotz Y. (2020). An Aging-Related Single-Nucleotide Polymorphism is Associated With Altered Clinical Outcomes and Distinct Inflammatory Profiles in Aged Blunt Trauma Patients. Shock.

[45] Gui W., Qiu C., Shao Q., Li J. (2021). Associations of Vascular Risk Factors, APOE and TOMM40 Polymorphisms With Cognitive Function in Dementia-Free Chinese Older Adults: A Community-Based Study. Front. Psychiatry.

[46] Talmud P.J., Drenos F., Shah S., Shah T., Palmen J., Verzilli C., Gaunt T.R., Pallas J., Lovering R., Li K. (2009). Gene-centric association signals for lipids and apolipoproteins identified via the HumanCVD BeadChip. Am. J. Hum. Genet..

[47] Clark D., Skrobot O.A., Adebiyi I., Susce M.T., de Leon J., Blakemore A.F., Arranz M.J. (2009). Apolipoprotein-E gene variants associated with cardiovascular risk factors in antipsychotic recipients. Eur. Psychiatry.

[48] Weidmann S. (1952). The electrical constants of Purkinje fibres. J. Physiol..

[49] Baruteau A.E., Probst V., Abriel H. (2015). Inherited progressive cardiac conduction disorders. Curr. Opin. Cardiol..

[50] Kléber A.G., Rudy Y. (2004). Basic mechanisms of cardiac impulse propagation and associated arrhythmias. Physiol. Rev..

[51] Michela P., Velia V., Aldo P., Ada P. (2015). Role of connexin 43 in cardiovascular diseases. Eur. J. Pharmacol..

[52] Del Ry S., Moscato S., Bianchi F., Morales M.A., Dolfi A., Burchielli S., Cabiati M., Mattii L. (2015). Altered expression of connexin 43 and related molecular partners in a pig model of left ventricular dysfunction with and without dipyrydamole therapy. Pharmacol. Res..

[53] Iwasaki Y.K., Nishida K., Kato T., Nattel S. (2011). Atrial fibrillation pathophysiology: Implications for management. Circulation.

[54] Makita N., Seki A., Sumitomo N., Chkourko H., Fukuhara S., Watanabe H., Shimizu W., Bezzina C.R., Hasdemir C., Mugishima H. (2012). A connexin40 mutation associated with a malignant variant of progressive familial heart block type I. Circ. Arrhythm. Electrophysiol..

[55] Lampe P.D., Lau A.F. (2004). The effects of connexin phosphorylation on gap junctional communication. Int. J. Biochem. Cell Biol..

[56] Rohr S., Kucera J.P., Kléber A.G. (1998). Slow conduction in cardiac tissue, I: Effects of a reduction of excitability versus a reduction of electrical coupling on microconduction. Circ. Res..

[57] Cole W.C., Picone J.B., Sperelakis N. (1988). Gap junction uncoupling and discontinuous propagation in the heart. A comparison of experimental data with computer simulations. Biophys. J..

[58] Boengler K., Schulz R., Heusch G. (2006). Connexin 43 signalling and cardioprotection. Heart.

[59] van Rijen H.V., Eckardt D., Degen J., Theis M., Ott T., Willecke K., Jongsma H.J., Opthof T., de Bakker J.M. (2004). Slow conduction and enhanced anisotropy increase the propensity for ventricular tachyarrhythmias in adult mice with induced deletion of connexin43. Circulation.

[60] Agullo-Pascual E., Cerrone M., Delmar M. (2014). Arrhythmogenic cardiomyopathy and Brugada syndrome: Diseases of the connexome. FEBS Lett..

